# Dynamics of the Myoglobin/Heme Complex: A Limited Proteolysis Perspective

**DOI:** 10.1021/acsmeasuresciau.6c00217

**Published:** 2026-08-10

**Authors:** Carter Lantz, Leila Minian, Roza Avetisyan, Zhenyu Xi, David H. Russell

**Affiliations:** Department of Chemistry, 14736 Texas A&M University, College Station, Texas 77843, United States

**Keywords:** limited proteolysis, mass spectrometry, ion mobility, average charge state, hydration, buffer molecules

## Abstract

The dynamics and stabilities (i.e., physicochemical properties) of proteins and protein complexes are dictated by the solution environment. Changes in the solution environment may alter the distribution of states present in solution (i.e., the free-energy landscape) and thus their physicochemical properties. This work examines the effects of temperature (4 °C, 21 °C, and 37 °C), electrospray ionization buffers (ammonium acetate (AmAc) and triethylammonium acetate (TEAA)), and solvent (H_2_O and D_2_O) on the physicochemical characteristics of the myoglobin/heme complex using limited proteolysis (i.e., trypsin digestion) and ion mobility-mass spectrometry (IM-MS). Electrospray ionization mass spectra of peptides formed by limited proteolysis showed increased peptide abundances at higher temperatures and in the absence of buffer molecules relative to the intact precursors. Interestingly, peptide abundances in D_2_O are more consistent across the temperature range studied relative to the peptide abundances in H_2_O, which demonstrates that D_2_O not only stabilizes proteins but acts as a kinetic trap for specific protein conformations. As an example, buffer components and D_2_O stabilize the myoglobin/heme interaction, as is evidenced by the modulation of peptide 64–77, an α-helical region that interacts with heme, and by shifts in the abundances of unfolded protein precursors. Lastly, IM-MS analysis of digested peptides suggests that AmAc alters the tertiary structure of the myoglobin/heme complex, whereas TEAA alters the secondary structure of the complex. The preservation of secondary structure in TEAA is further illustrated by the shift in arrival time distributions of 1+ ions in TEAA relative to the 2+, 3+, 4+, and 5+ ions present in other buffer conditions. Overall, the data show that buffer-solvent interactions govern the hydration of myoglobin and consequently, the physicochemical properties of the complex.

## Graphical abstract

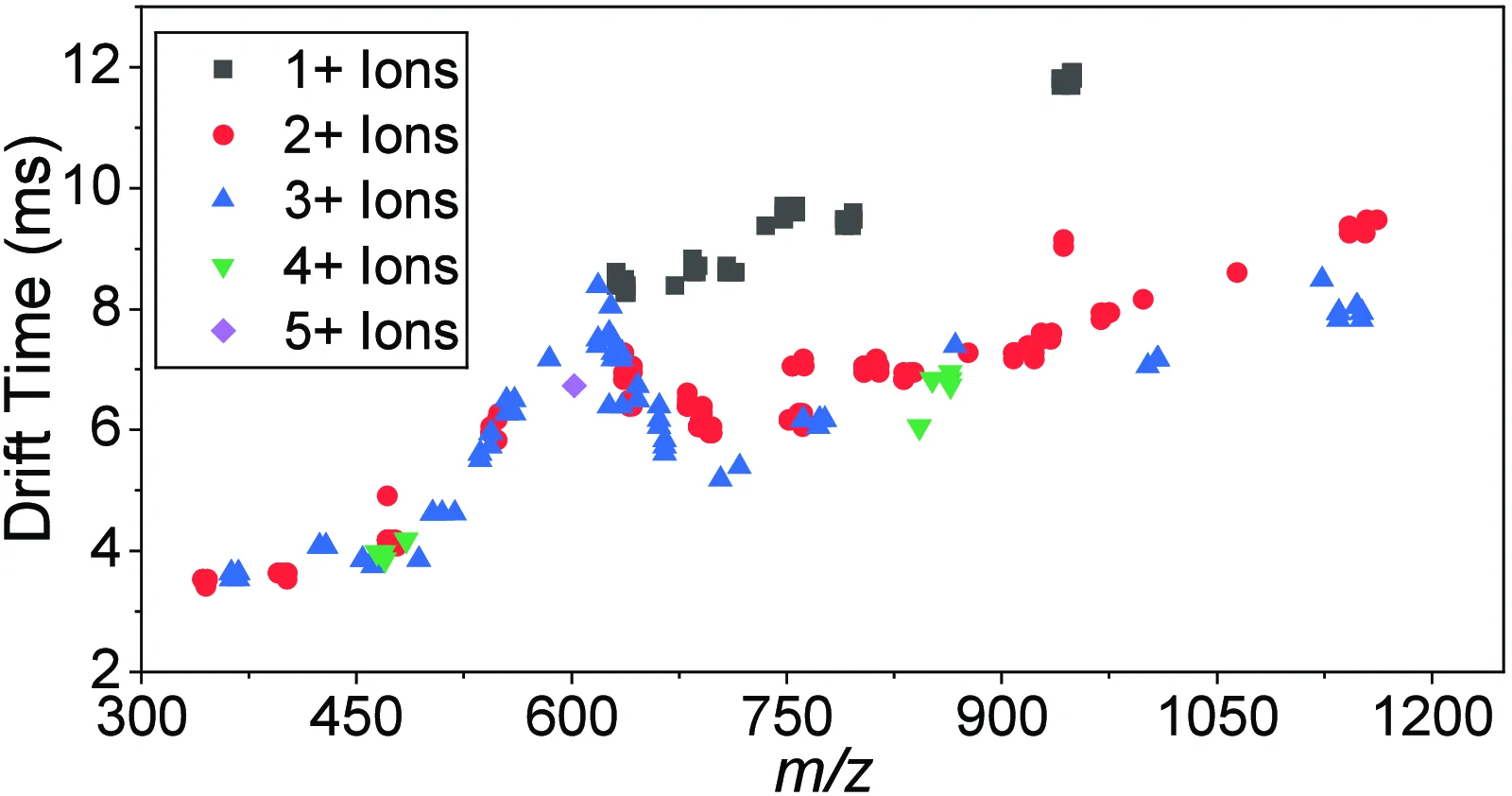

## Introduction

Proteins and protein complexes exist as conformational ensembles that form a free-energy landscape (FEL), where states are distinguished by distinct dynamics and stabilities.^1–6^ It is thought that hydration dictates the FEL of proteins and protein complexes; however, that view is controversial, and the extent of hydration dictating protein FELs is unclear.^7–10^ Marques et al. reported that protein dynamics are not slaved to hydration,^7^ whereas Frauenfelder et al. argued that protein dynamics are slaved to hydration.^8^ Zhao et al. reported molecular dynamics simulations that demonstrate that water plays a “dominant” role in dictating barstar conformational entropy.^9^ Tilton et al. demonstrated using X-ray crystallography that slight changes in structure are due, in large part, to shifts in solvent interactions.^10^ More-recent native mass spectrometry data provide insight into how environmental factors such as temperature, the presence of osmolytes, and solvent composition shift the distribution of states present in solution.^11–15^ Shifts in solution temperature have been found to alter the dynamics of protein complexes. At high temperatures, proteins are restructured by shifts in hydrogen bonding and alterations can result from interactions with salt molecules.^16,17^ At cold temperatures, proteins are restructured when water molecules hydrate hydrophobic regions of proteins.^17,18^ The presence of osmolytes and buffer molecules in protein solutions has also been found to shift their dynamics and stabilities.^19–22^ It has been hypothesized that osmolytes alter the dynamics of proteins and protein complexes by shifting their hydration.^12,15,23^ In addition, recent studies on protein dynamics in deuterium oxide (D_2_O) have provided evidence that proteins behave differently in D_2_O compared to H_2_O, because of hydration shifts.^14,24–26^ Some studies have reported that in D_2_O, proteins become rigid due to altered hydration of the complex.^25,27^ Shifts in hydration with D_2_O can also influence key protein mechanisms such as aggregation of collagen.^28,29^ If shifts in the FEL can be monitored, a greater amount of information on protein mechanisms and their physicochemical properties may be obtained.

Limited proteolysis involves the cleavage of protein covalent bonds with low concentrations of protease enzymes. Analysis of the resulting peptide ions can yield not only sequence information but also structural, dynamics, and stability information on proteins and protein complexes. Protease enzymes have been found to readily cleave disordered or loop regions of proteins, so peptides resulting from limited proteolysis can inform on the flexible regions of proteins.^30,31^ We have determined that limited proteolysis can provide information on shifts in cytochrome *c* and myoglobin structures in different solvents, when the protein is thermally denatured, or when the protein is phosphorylated.^32–35^ In addition, limited proteolysis has been utilized to obtain information on shifts in protein structure upon ligand binding.^36,37^ Our previous research using matrix-assisted laser desorption ionization determined that that 1+ peptides retain structure information after digestion and ionization.^38^ Others have determined that higher-charged ions generated using electrospray ionization have distinctly different drift times, relative to 1+ ions, which indicates that ions with higher charge have different structures.^39^ Recently, limited proteolysis has been used to analyze the structure of proteins on a proteome-wide scale.^40–42^ These studies establish the use of limited proteolysis as a tool to obtain information on the structure, dynamics, and stabilities of protein complexes and can therefore provide insights into protein physicochemical properties and the FEL.

It is difficult to determine the roles of the solution environment including hydration on the physicochemical properties of protein complexes; however, methods are being developed that provide insight into these important factors. In this work, we analyze myoglobin dynamics and stabilities by utilizing limited proteolysis combined with ion mobility–mass spectrometry (IM-MS). Myoglobin was chosen for this study because of its α-helical nature and its interaction with a heme molecule.^32,33,43,44^ Previous research has determined that peptides retain their helical structure after digestion when transferred into the gas phase.^38,45^ As a result, myoglobin is a protein standard that can be used to provide information on how α-helices react to changes in the solution environment. The information from limited proteolysis of myoglobin provides relevant information on the myoglobin heme complex and provides insight into how the solution environment alters the dynamics of myoglobin and the stabilities of the myoglobin/heme complex.

## Materials and Methods

### Sample Preparation

Myoglobin was purchased from Sigma–Aldrich and dissolved in H_2_O or D_2_O solutions. Trypsin was purchased from Thermo Fisher, and it was dissolved in H_2_O at a concentration of 2 μM and stored at −80 °C. Ammonium acetate (AmAc) and triethylammonium acetate (TEAA) were purchased from Sigma–Aldrich. 75 nM trypsin was then added to 15 μM samples of myoglobin. Solutions containing buffer molecules were prepared following the same process, with the addition of either 200 mM AmAc or 200 mM TEAA. For solutions containing no buffer molecules, a D_2_O percentage of 96% was used, and for solutions containing buffer molecules, a D_2_O percentage of 75% was used so the concentration of buffer molecules and trypsin remained the same throughout the experiments. H_2_O and D_2_O solutions were allowed to incubate at ambient temperature (21 °C), 4 °C, or 37 °C for 24 h before electrospray ionization mass spectrometry analysis.

### Ion Mobility–Mass Spectrometry Experiments

Upon completion of limited proteolysis, samples were loaded into pulled gold-coated electrospray capillaries, pulled using a Sutter Instruments Model P-1000 Micropipette Puller. The capillaries were electrosprayed in positive ion mode on a Waters Synapt G2 instrument in mobility TOF mode. The capillary voltage parameter ranged from 1.0 to 1.5 kV, the sampling cone was set at 40 V, the extraction cone voltage was set at 1.0 V, and the source temperature was kept at 25 °C. Nitrogen was used as the drift gas. Mass spectra were collected for 10 min with scan times of 1 s.

### Data Analysis

Waters MassLynx software was used for the analysis of the mass spectra. An in-house script and pulsar software were used to process and analyze peptide ion mobility data from 300 to 1250 *m*/*z*.^46^ The Expasy PeptideMass calculator was used to calculate peptide masses with up to 5 missed cleavages.^47^ Peptide identities were verified by comparing experimental *m*/*z* values with computed theoretical masses, and charge states were assigned depending on their isotopic spacing and *m*/*z* distribution patterns. Mass spectra were normalized using the raw intensity from summed mobilograms. Drift times for each peptide were recorded after applying 2 smooths with a window size of 5. The structure of myoglobin displayed corresponds to PDB code 1WLA.^48^

## Results

Proteins have numerous macrostates and microstates in solution, and probing these states can provide insights into their dynamics and stabilities (i.e., physicochemical properties). Shifts in the solution environment (i.e., temperature, buffer molecules, solvent, and osmolytes) alter the distribution of protein states in solution. To determine how solution environmental factors influence the physicochemical properties of protein/ligand complexes, we subjected the myoglobin/heme complex to limited proteolysis with trypsin under different solution conditions. The data demonstrate that both buffer molecules and solvents (H_2_O and D_2_O) play roles in dictating hydration of the myoglobin/heme complex and thus its physicochemical properties.

### Shifts in Solution Chemistry Alter Myoglobin Peptide Abundance

To provide information on how buffer molecules alter the physicochemical properties of myoglobin, limited proteolysis was performed in H_2_O solutions under these conditions: (1) no buffer present; (2) with AmAc as a buffer; and (3) with TEAA as a buffer (Figure 1). In each mass spectrum, peptides and intact precursors are present, so it is possible to compare the digestion efficiency of myoglobin by trypsin in each solution. When the solution was incubated at 4 °C with no buffer molecules, peptide abundance is low, relative to the intact precursors, but peptide abundance increases when the solution temperature is increased to 21 °C and increases further when the solution temperature is 37 °C. When AmAc buffer molecules are present in solution, peptide abundance decreases relative to the abundance of peptides in solutions that do not contain buffer molecules at each temperature value, which demonstrates that AmAc buffer molecules decrease the efficiency of myoglobin digestion. Solutions containing TEAA also show decreases in peptide abundance relative to solutions with no buffer molecules at each solution temperature, which demonstrates that the presence of TEAA buffer molecules also reduces the digestion efficiency of myoglobin. The reduced peptide abundance in solutions containing buffer molecules indicates that the buffer stabilizes the myoglobin/heme complex and inhibits tryptic digestion of the intact precursors.

**Figure 1:**
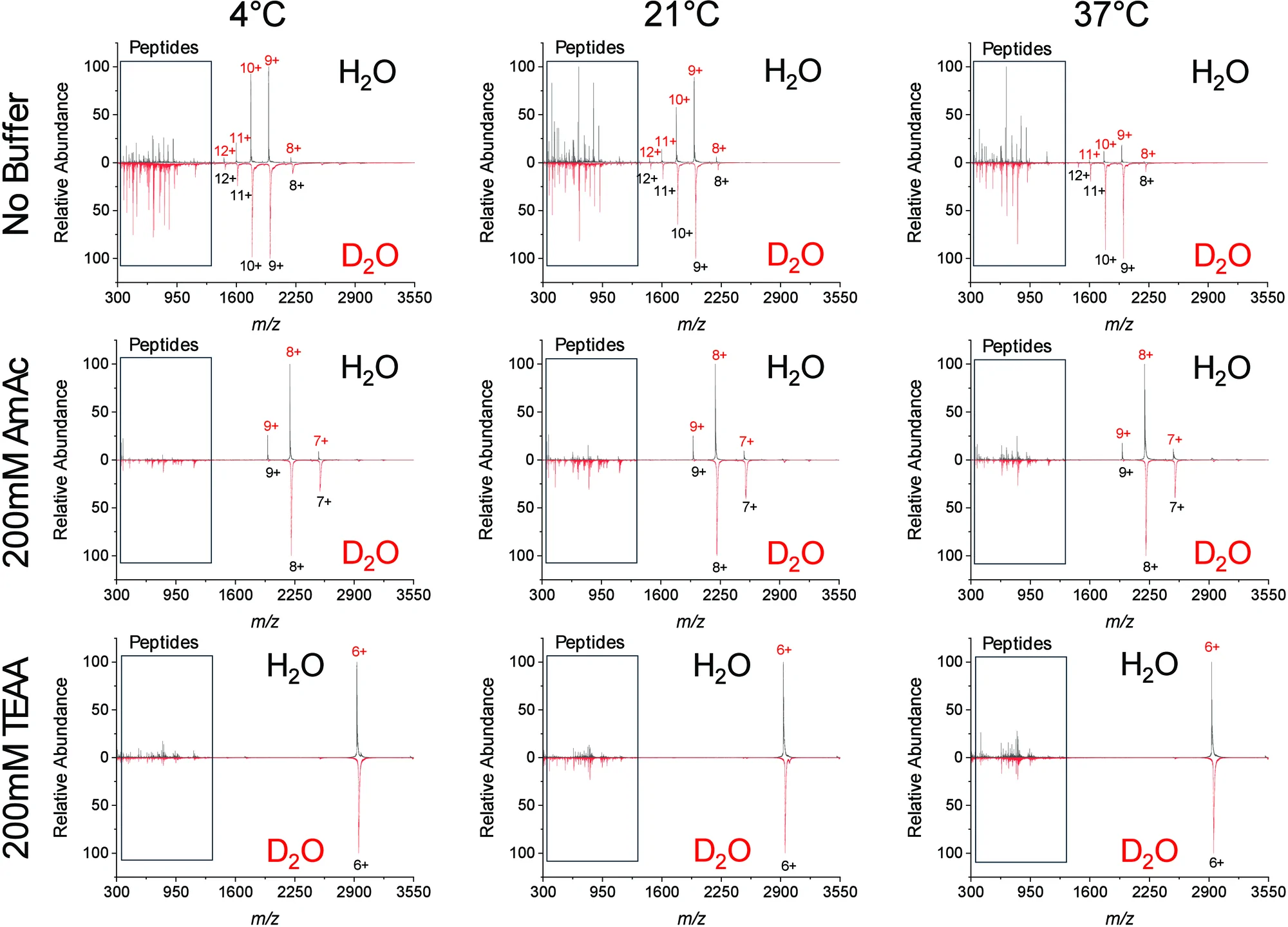
Mass spectra of myoglobin digestion reactions in H_2_O and D_2_O solutions at 4 °C, 21 °C, and 37 °C containing no buffer molecules, 200 mM AmAc, and 200 mM TEAA. Notice the difference in relative abundance of the peptides, relative to the intact precursors.

Buffer molecules also alter the physicochemical properties of myoglobin when trypsin digestion is performed in D_2_O. Similar to the spectra in H_2_O, each mass spectrum of D_2_O solutions contained both peptides and intact precursors, making it possible to directly compare the digestion efficiency of myoglobin by trypsin across these two solvents. In digestion experiments performed in D_2_O without buffer molecules, abundant peptides and precursor signals are present in the mass spectra at each temperature (Figure 1). Notably, the abundance of peptides relative to the intact precursors in D_2_O is more consistent across the 4–37 °C temperature range, compared to the peptide abundance in H_2_O. When AmAc molecules were present in solution, peptide abundance was lower over the temperature range, compared to the abundance in solutions without buffer molecules. This is evidence that AmAc molecules reduce the efficiency of myoglobin digestion in D_2_O. The same trend was observed with TEAA as a buffer, which is evidence that TEAA molecules also reduce the efficiency of myoglobin digestion in D_2_O. The significant decrease in peptide abundance when buffer molecules are present in solution is evidence that buffer molecules have a greater effect on the dynamics and stabilities of myoglobin than D_2_O alone. Addition of buffer molecules into solutions containing the myoglobin heme complex decreases the efficiency of tryptic digestion of myoglobin, which demonstrates that buffer molecules contribute to complex physicochemical properties.

### Peptide Abundance Reveals How Solution Conditions Alter Myoglobin Dynamics

Analysis of specific peptide abundances reveals that the dynamics and stabilities of the myoglobin/heme complex shift when solution conditions are altered. When digestion is performed in H_2_O with no buffer molecules, peptide 1–31, an N-terminal peptide, remains abundant across the temperature range (Figure 2). In D_2_O, the abundance of peptide 1–31 decreases with an increase in temperature demonstrating that the dynamics of the N-terminus is sensitive to the presence of D_2_O. When AmAc and TEAA buffer molecules are added to the solution, peptide 1–31 is not observed at any temperature in H_2_O or D_2_O. It is notable that peptide 17–31, a part of peptide 1–31, is also present in this region of the mass spectrum. When buffer molecules are absent in solution, the abundance of peptide 17–31 decreases readily in H_2_O at higher temperatures in solutions without buffer molecules; however, the abundance of the peptide increases at higher temperatures in D_2_O, which is further evidence that the N-terminus of myoglobin is sensitive to the presence of D_2_O. When buffer molecules are present in solution, peptide 17–31 is the base peak in the spectra, which is evidence that peptide 17–31 is readily cleaved even when buffer molecules are present in solution. Abundance analysis of peptide 1–31 and peptide 17–31 reveals that the presence of buffer molecules and D_2_O alters the N-terminal dynamics of myoglobin.

**Figure 2:**
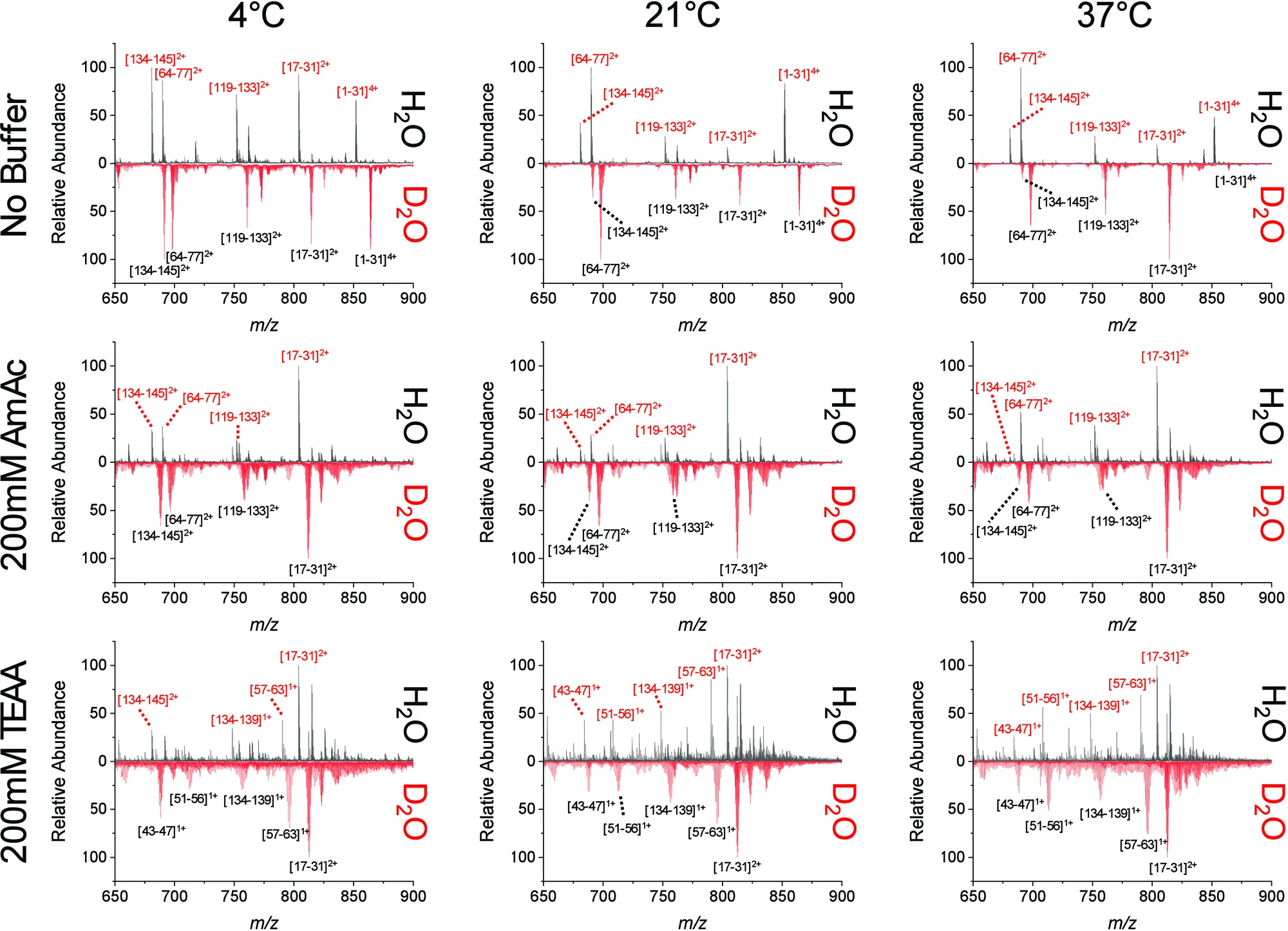
Mass spectra of myoglobin peptides in H_2_O and D_2_O solutions at 4, 21, and 37 °C containing no buffer molecules, 200 mM AmAc, and 200 mM TEAA. Notice the difference in relative abundance of the peptides relative to that of other peptides in the mass spectra.

Another peptide that is present in many of the solutions in this study is peptide 64–77. When buffer molecules are absent in H_2_O solutions, peptide 64–77 increases in abundance relative to the other peptides in the spectrum when the solution temperature is increased from 4 to 21 °C and the abundance further increases when the solution temperature is increased to 37 °C. When buffer molecules are absent in D_2_O solutions, there is a rise in relative abundance of peptide 64–77 when the solution temperature is increased from 4 to 21 °C, but a decrease in relative abundance of this peptide when the solution is increased from 21 to 37 °C. The difference in abundance of peptide 64–77 in D_2_O relative to H_2_O shows that the dynamics of this region of myoglobin is sensitive to the presence of D_2_O. When AmAc is added to the solution, peptide 64–77 decreases in relative abundance in both H_2_O and D_2_O. Peptide 64–77 is largely absent from solutions containing TEAA; however, peptides corresponding to 57–63 and 51–56 are present. Shifts in abundance of peptide 64–77 is evidence that temperature, buffer molecule composition, and D_2_O alter the dynamics and stabilities of the myoglobin/heme complex, and thus the cleavage of peptide 64–77.

Analysis of peptides resulting from limited proteolysis provides information on the C-terminal region of myoglobin. The abundance of peptide 119–133 is present in high abundance at 4 °C in both H_2_O and D_2_O solutions where buffer molecules are absent, but its abundance decreases relative to the other peptides when the temperature of H_2_O and D_2_O solutions are increased to 21 and 37 °C; however, the shift in abundance is muted in D_2_O. Similarly, peptide 134–145 increases in abundance at lower temperatures, relative to the other peptides in the spectrum in both H_2_O and D_2_O. When AmAc buffer molecules are present in H_2_O and D_2_O solutions, peptides 119–133 and 134–145 are present, but their abundance decreases relative to the abundance of the peptides in solutions without buffer molecules. Peptide 119–133 is completely absent in solutions containing TEAA at all temperatures. Peptide 134–145 is also largely absent, but a peptide corresponding to 134–139 is present. Analysis of peptide 119–133 and 134–145 under different buffer conditions shows that the C-terminus of myoglobin is also sensitive to shifts in solution chemistry.

### Buffer Molecules Alter Myoglobin Peptide Mobility

IM-MS provides further information on the dynamics and stabilities of peptides that result from limited proteolysis. IM-MS was performed on digested peptides in 21 °C solutions of H_2_O and D_2_O that did not contain buffer molecules (see Figure 3A, as well as Figure S1A and Tables S1 and S2), solutions containing AmAc (see Figure 3B, as well as Figure S1B and Tables S3 and S4), and solutions containing TEAA (see Figure 3C as well as Figure S1C and Tables S5 and S6). Arrival time distribution (ATD) profiles demonstrate that peptide mobility does not shift in D_2_O, compared to H_2_O, but the profiles do shift in the presence of certain buffer molecules. ATD profiles are similar in both the absence of buffer molecules (Figure 3A) and in AmAc (Figure 3B). Namely, the plots show the presence of small peptides in *m*/*z* region 300–500 with arrival times between 4 and 5 ms, the presence of peptides in *m*/*z* region 500–700 with drift times between 5 and 10 ms, and the presence of peptides in the *m*/*z* region 700–1200 with drift times of 6 to 8 ms. The *m*/*z* vs arrival time plots for solutions containing no buffer molecules also show the presence of intact precursors in *m*/*z* region 700–1200 with drift times between 7 and 10 ms (vide infra). When TEAA molecules are present in solution, the peptide mobilities in the *m*/*z* vs arrival time plots shift (Figure 3C). In the TEAA ATD plots there is a population of abundant singly charged peptides in the *m*/*z* region 500–1000 with drift times corresponding to 8 to 12 ms, and there is a population of doubly charged peptides in the *m*/*z* region 800–1200 with drift times corresponding to 6 to 10 ms. Shifts in peptide mobility when TEAA molecules are present in solution illustrates that TEAA alters myoglobin dynamics and stabilities. We interpret the differences in both peptide identity and peptide arrival time in the different solutions as evidence that buffer molecules alter the FEL of myoglobin and thus shift myoglobin physicochemical properties.

**Figure 3:**
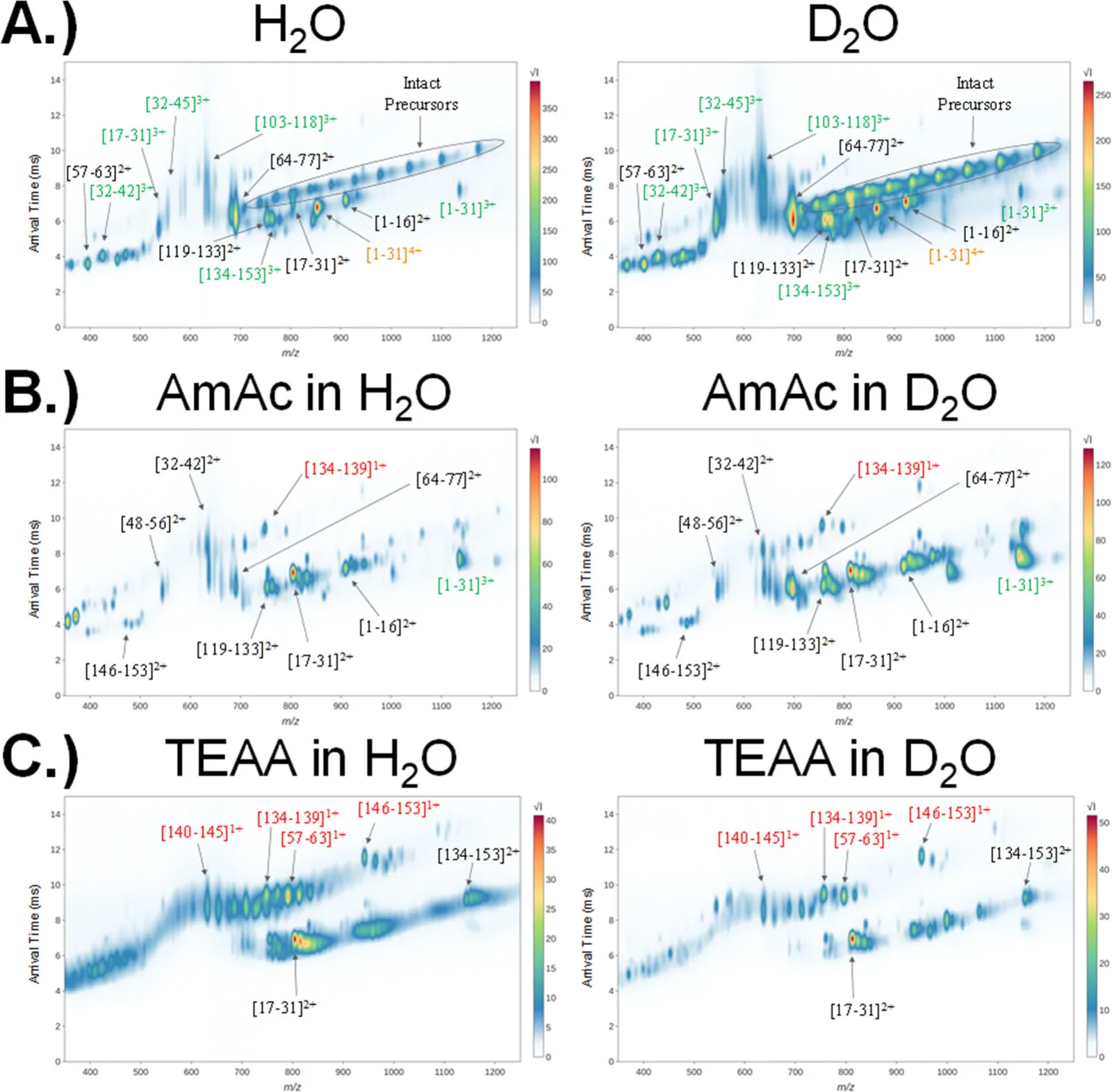
*M/z* vs arrival time plots of myoglobin digestion products in H_2_O and D_2_O solutions containing (A) no buffer molecules, (B) 200 mM AmAc, and (C) 200 mM TEAA. Notice the difference in peptide arrival time pattern between the three buffer conditions.

It is noteworthy that the mobiligrams at 4 and 37 °C are similar to the mobiligrams at 21 °C; however, there are a couple of exceptions. At 4 °C, the intact precursors are more abundant in H_2_O compared to D_2_O (Figure S2A and Tables S7 and S8). In AmAc at 4 °C, the abundance of the 1+ ions was lower in H_2_O relative to their abundance at 21 °C (Figure S2B and Tables S9 and S10). In TEAA at 4 °C, the C-terminal peptide 134–145 is more abundant compared to the abundance of the peptide 21 °C (Figure S2C and Tables S11 and S12). At 37 °C, intact precursor signals are readily apparent in D_2_O but are largely absent in H_2_O (Figure S3A and Tables S13 and S14). It is also interesting to note that peptides digested at 37 °C in H_2_O and D_2_O have a greater full width at half maximum (fwhm), relative to the peptides at 4 and 21 °C (Figure S3A). In AmAc (Figure S3B and Tables S15 and S16) and TEAA (Figure S3C and Tables S17 and S18) at 37 °C, the fwhm of the peptides do not shift, compared to their mobilities at 21 °C, which is further evidence that buffer molecules alter myoglobin dynamics. These findings reveal that variations in temperature can shift the physicochemical properties of the myoglobin/heme complex, which can account for the altered digestion of myoglobin at the different temperatures used in this study.

## Discussion

The limited proteolysis experiments performed demonstrate that changes in the solution environment (e.g., temperature, buffer conditions, and solvent conditions) shift the FEL of the myoglobin/heme complex and thus its physicochemical properties. Shifts in peptide abundances relative to the intact precursor provide information on complex dynamics (Figure 1). Shifts in peptide abundance relative to other peptides provide information about the dynamics of specific myoglobin regions and inform on the stabilities between myoglobin and the heme molecule (Figure 2). Furthermore, ion mobility provides information on how changes in the solution environment shift myoglobin tertiary and secondary structure (Figure 3). Overall, these analyses provide detailed insights into shifts of the myoglobin FEL and highlight how hydration dictates the myoglobin physicochemical properties.

Changes in the solution environment shift hydration of the myoglobin/heme complex, which alters its dynamics. Figure 1 reveals that as the temperature increases, the efficiency of digestion is increased, which is evidence that higher temperatures increase the dynamics of the myoglobin/heme complex. Previous studies have demonstrated that shifts in temperature alter hydration of proteins, thereby shifting protein physicochemical properties.^11,17,18,49–52^ So, we attribute increases in myoglobin dynamics at higher temperatures as evidence that shifts in hydration alter myoglobin physicochemical properties. Conversely, the addition of buffer molecules decreases the abundance of peptides in the solution, demonstrating that buffer molecules reduce the dynamics of the myoglobin/heme complex. Recent studies have provided evidence that buffer molecules act as chaotropes or kosmotropes by directly interacting with the protein and altering its hydration.^13–15,21,53,54^ Therefore, we attribute decreases in peptide abundance when buffer molecules are present as evidence that AmAc and TEAA act as kosmotropes by interacting noncovalently with myoglobin, which shifts hydration of the complex and alters its physicochemical properties. The data presented show that solution temperature and buffer composition have a significant effect on myoglobin hydration, resulting in altered myoglobin dynamics.

It could be argued that shifts in peptide abundance in Figure 1 are a result of shifts in trypsin efficiency in the different solutions rather than shifts in the myoglobin dynamics. Although it has been extensively reported that shifts in environmental conditions shift the activity of trypsin,^55–57^ Figure 2 illustrates that shifts in peptide abundance are not solely due to shifts in trypsin activity. In Figure 2, specific peptide abundances shift in response to differences in the solution temperature, buffer, and solvent. If the difference in peptide abundance in Figure 1 were due primarily to trypsin activity, the relative abundance of peptides in Figure 2 should remain the same across solution conditions. Clearly, the abundance of peptides in Figure 2 differs in the different solutions, so the abundance of peptides reported in Figure 1 is, in part, due to the dynamics of myoglobin. Because peptide abundances are reflective of myoglobin dynamics, we can use the individual peptide abundances to make claims about how different environmental conditions alter the dynamics of the complex.

Analysis of specific myoglobin peptide abundances shows that the dynamics of myoglobin shift with changes in the solution environment. Peptides 1–31 and 17–31 are cleaved readily in solutions with no buffer and solutions that contain AmAc, which demonstrates that the N-terminus of myoglobin is more solvent accessible than other regions of the protein. Peptides 1–31 and 17–31 are also more readily cleaved by trypsin at high temperatures, showing that the N-terminal region of myoglobin is sensitive to thermal restructuring (see Figure 4, as well as Figure S4). Thermal restructuring occurs before complete unfolding of the protein and is often associated with changes in complex hydration and shifts in protonation microstates.^17,58^ On the C-terminus of myoglobin, peptide 119–133 and peptide 134–145 decrease in abundance when buffer molecules are included into solution, suggesting that buffer molecules readily interact with the C-terminus of myoglobin (see Figure 4 and Figure S4). In addition, peptide 119–133 and peptide 134–145 are more readily cleaved at lower temperatures relative to other peptides, which corresponds to non-Arrhenius behavior. The non-Arrhenius behavior observed in the digest is evidence that the C-terminus of myoglobin is sensitive to cold restructuring (i.e., when water molecules intercalate into hydrophobic areas of the protein).^17,18^ Shifts in peptide abundance as a function of temperature and buffer molecule composition suggest that hydration dictates the dynamics of myoglobin in solution.

**Figure 4:**
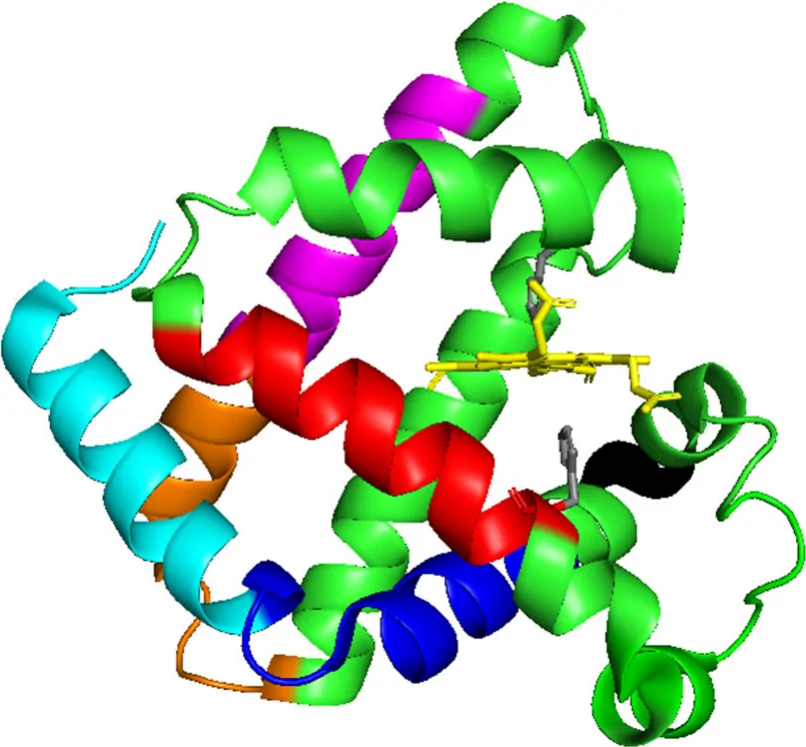
A structure of the myoglobin/heme complex with peptide 1–16 (light blue), peptide 17–31 (dark blue), peptide 64–77 (red), peptide 119–133 (orange), and peptide 134–145 (purple) labeled. The heme molecule is highlighted in yellow, and the histidine residues that interact with it are shown in gray. A kinked α-helix responsible for elevated drift times is labeled in black.

Analysis of peptide abundance also provides information about the stabilities of the myoglobin/heme complex. Peptide 64–77 corresponds to a region of myoglobin that contains a histidine residue that interacts with heme (Figure 4 and Figure S4). Peptide 64–77 is more abundant at higher temperatures, which is evidence that the interaction between myoglobin and heme is sensitive to thermal restructuring and is sensitive to hydration. When buffer molecules are present, peptide 64–77 decreases in abundance, which is evidence that buffer molecules stabilize the myoglobin/heme complex. We interpret the increased stability of peptide 64–77 in the presence of buffer molecules as evidence that buffer components interact with myoglobin, resulting in a change in hydration of the complex that stabilizes the interaction between myoglobin and heme.

Peptide mobility demonstrates that AmAc buffer molecules alter the tertiary structure of myoglobin and TEAA alters the secondary structure of myoglobin α-helices. Previous research has determined that the helical structure of peptides is preserved when transferred into the gas phase for ion mobility analysis,^38,45^ so the shape of the mobiligrams informs on the helical structures of myoglobin. The shape of the *m*/*z* vs arrival time plots in the solutions without buffer molecules (Figure 3A) and the solutions containing AmAc (Figure 3B) are similar, although the peptide abundance decreases when AmAc is present (Figure 1). The observation that the *m*/*z* vs arrival time plots are similar even though the peptide abundance has decreased is evidence that the dynamics of the myoglobin helices (i.e., secondary structure) are not shifted by AmAc, but the connectivity between the helices (i.e., tertiary structure) is shifted. On the other hand, the shape of the *m*/*z* vs arrival time plots shifts in TEAA, relative to the other two buffers (Figure 3C). The altered shape of the *m*/*z* vs arrival time plots and the decrease in peptide abundance indicate that TEAA shifts the dynamics of myoglobin helices (i.e., secondary structure). Shifts in myoglobin secondary structure by TEAA is supported by data in Figure 2, which shows that, in TEAA, peptides 64–77 and 134–145 are absent but related peptides 57–63, 51–56, and 134–139 are present. TEAA interacts differently with proteins relative to AmAc, because it is more hydrophobic and has a different basicity,^15^ so it is possible that there is a difference in the way TEAA and AmAc restructures the hydration of myoglobin that affects its secondary structure.

Peptide mobility data also reveals that certain regions of myoglobin have high conformational entropy. The peptide region between 500 and 700 *m*/*z* in solutions electrosprayed without buffer (see Figures S1A, S2A, and S3A) and with AmAc (Figures S1B, S2B, and S3B) have elevated and broad arrival times, even though the charge for the peptides is 2+, 3+, and 5+. In the crystal structure of myoglobin, the broad signals with elevated arrival times correspond to a kinked α-helical region (amino acids 36–37) of the protein and helices 5 (amino acids 60–79), 6 (amino acids 83–97), and 7 (amino acids 101–119) (see Figure 4 and Figure S4). The elevated arrival times of the peptides corresponding to the kinked α-helix is evidence that elements of the kinked α-helix are preserved once the peptide is digested. The elevated arrival times of peptides corresponding to helix 5, helix 6, and helix 7 demonstrate that these helices have high conformational entropy and may retain their helical structure. The arrival times in regions 500–700 *m*/*z* provide evidence that digested peptide dynamics and stabilities resemble those of intact myoglobin and can inform on its physicochemical properties.

It has been reported that proteins behave differently in D_2_O, compared to H_2_O;^24,25,27,59^ the limited proteolysis experiments performed in this study support this claim. The data show that peptide abundances remain similar across a temperature range of 4–37 °C in D_2_O, relative to peptide abundances in H_2_O across the same temperature range (Figure 1). Proteins are more rigid in D_2_O relative to H_2_O because of shifts in hydration,^27^ so the similar abundance of peptides in D_2_O across temperature is evidence that D_2_O acts as a kinetic trap for myoglobin conformations. Figure 2 demonstrates that abundances of peptides 1–31 and 17–31 are shifted in D_2_O compared to H_2_O but peptides 134–145 and 119–133 do not shift significantly, which is evidence that the N-terminus is more sensitive to hydration with D_2_O compared to the C-terminus (Figure 4; Figure S4). In addition, peptide 64–77 is less abundant in D_2_O relative to its abundance in H_2_O at higher temperatures, which is evidence that hydration of myoglobin with D_2_O stabilizes its interaction with heme (Figure 4 and Figure S4). Even though peptide abundances in D_2_O change compared to H_2_O, the shapes of the mobiligrams do not shift (Figure 3), which is evidence that D_2_O alters protein tertiary structure, but not its secondary structure. Shifts in peptide abundances with D_2_O reveal that hydration of myoglobin with D_2_O alters the FEL of the myoglobin/heme complex and thus its physicochemical properties.

Unfolded intact precursor abundances in the *m*/*z* vs arrival time plots further highlight the differences between H_2_O and D_2_O. When limited proteolysis reactions were incubated at 4 °C, intact precursor signals were more abundant in H_2_O rather than D_2_O (Figure 5A and Figure S1A). The increased abundance of intact precursor signals in H_2_O is evidence that myoglobin is more susceptible to cold restructuring at 4 °C in H_2_O compared to D_2_O. In solutions containing no buffer molecules at 21 °C, intact precursor signals are more abundant in D_2_O compared to their abundance in H_2_O (Figure 5B and Figure S2A). The increase in abundance of precursor signals in D_2_O is evidence that myoglobin is more likely to restructure in D_2_O relative to H_2_O at 21 °C. When the solution temperature of the reaction increases to 37 °C, intact precursor signals are far less abundant in H_2_O than they are in D_2_O (Figure 5C; Figure S3A). The presence of intact precursor signals in D_2_O but not in H_2_O is evidence that myoglobin is more susceptible to heat restructuring in D_2_O rather than H_2_O. We attribute differences in precursor abundance as a function of temperature as evidence that H_2_O and D_2_O hydrate myoglobin differently. Since H_2_O and D_2_O have different properties (i.e., viscosity, dielectric constant, and pH),^24,60^ hydration of myoglobin will shift. Shifts in myoglobin hydration with the different solvents will alter the physiochemical properties of the protein and thus the amount of protein unfolding at different temperatures.

**Figure 5:**
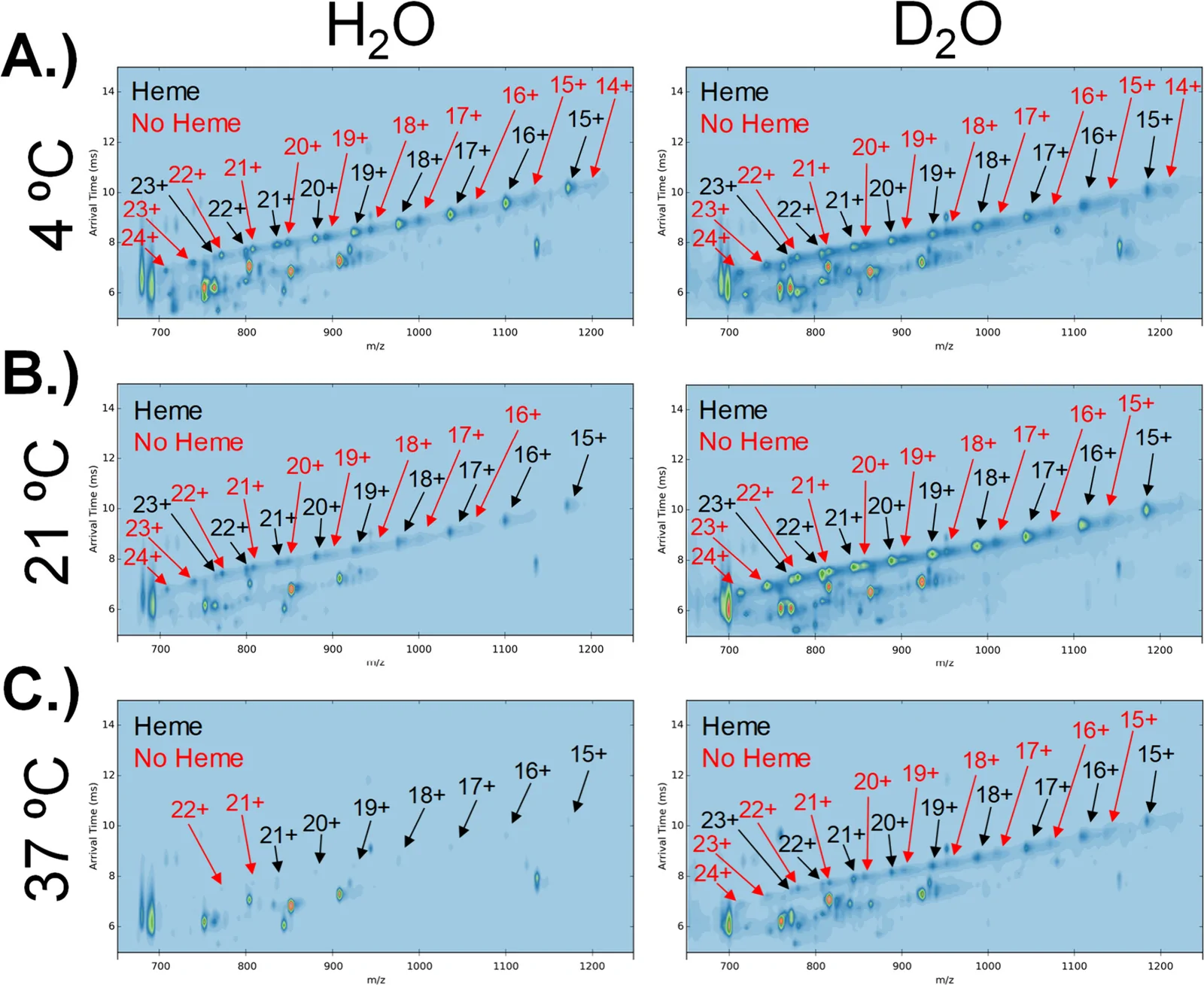
*M/z* vs arrival timeplots of myoglobin intact precursors in H_2_O and D_2_O solutions containing no buffer molecules when the reactions were incubated at (A) 4 °C, (B) 21 °C, and (C) 37 °C. The difference in abundance of the precursors in H_2_O and D_2_O at each temperature highlight the differences between H_2_O and D_2_O.

## Conclusion

Limited proteolysis results in this work highlight how both buffer molecules and solvent (H_2_O and D_2_O) play a role in dictating the dynamics and stabilities of the myoglobin/heme complex. It has been hypothesized that hydration dictates protein FELs, but the extent to which hydration governs protein FELs is debated.^7–10^ Our view is that hydration has a strong effect on protein physicochemical properties, but other considerations such as temperature, buffer molecules, and solvent composition also play key roles. When buffer molecules are not present in H_2_O solutions, peptide abundance increases as a function of temperature, which is interpreted as evidence that shifts in hydration alter the physicochemical properties of myoglobin. The presence of buffer molecules (AmAc and TEAA) decreases abundances of some peptides, which is evidence that buffer molecules alter the dynamics and stabilities of myoglobin. Alterations in myoglobin dynamics and stabilities are due to buffer molecules acting as kosmotropes by interacting noncovalently with myoglobin, rearranging hydration of the complex, and shifting its physicochemical properties.^13,15,53^ Peptide abundance is similar across the temperature range of 4 to 37 °C in the presence of D_2_O, compared to the peptide abundance in H_2_O. The muted shift in peptide abundance in D_2_O is evidence that alteration of hydration with D_2_O not only stabilizes proteins but acts as a kinetic trap for specific conformations.^27^ Furthermore, the claim that D_2_O acts as a kinetic trap is supported by the modulation of unfolded precursor abundances observed by IM-MS experiments. When both buffer molecules and D_2_O are present in solution, the cumulative effects of both are observed. Namely, peptide abundance decreases and is more consistent across temperature in buffered solutions containing D_2_O. The cumulative effects of buffer molecules and D_2_O demonstrate the intricate interplay between buffer and solvent to dictate hydration of the myoglobin/heme complex and thus its physicochemical properties.

Analysis of individual peptide abundances from limited proteolysis experiments provides more-detailed information on how buffer molecules and solvent (H_2_O and D_2_O) shift the FEL of myoglobin. Analysis of cleaved peptide abundances from myoglobin in H_2_O and D_2_O reveals that the N-terminus is more sensitive to shifts in hydration compared to the C-terminus. Furthermore, shifts in abundance of peptide 64–77, a peptide that interacts with the heme molecule, in H_2_O and D_2_O reveal that changes in hydration alter the myoglobin/heme interaction. Shifts in peptide abundance as a function of temperature, buffer, and presence of D_2_O are consistent with previous research by Giubertoni et al. and Spyrakis et al., highlighting the roles of hydration in protein dynamics and ligand binding.^24,61^ IM-MS, which reveals peptide dynamics information, provides information on shifts in myoglobin secondary and tertiary structure. AmAc molecules decrease the abundance of peptide ions, but not their shape suggesting that AmAc only alters the tertiary structure of the myoglobin/heme complex. TEAA molecules, on the other hand, alter both the shape and abundance of peptide ions, which is evidence that TEAA molecules alter the secondary structure of myoglobin α-helices in addition to the tertiary structure. Collectively, these results demonstrate that the interplay between buffer molecules and the solvent dictates protein FELs and physicochemical properties. Furthermore, they highlight the important role hydration plays in the stabilities of protein/ligand complexes.

## Supplementary Material



## Notes

Additional information including ion mobility experiments at different temperatures and tables with all identified peptides (PDF)
