# Supplementary material for: Dynamics of the Myoglobin/Heme Complex: A Limited Proteolysis Perspective
Source: ACS Meas Sci Au. 2026 Aug 10;6(5):1848–57. doi: 10.1021/acsmeasuresciau.6c00217 (PMC13693280; doi:10.1021/acsmeasuresciau.6c00217)
Supplement: Supplementary file 1 [file tg6c00217_si_001.pdf]

Supporting Information

Dynamics of the Myoglobin/Heme Complex: A Limited Proteolysis Perspective

Carter Lantz, Leila Minian, Roza Avetisyan, Zhenyu Xi, David H. Russell*
Department of Chemistry, Texas A&M University, College Station, TX 77843
*Corresponding author; Email: russell@chem.tamu.edu

Table of Contents

	Page
Figure S1	S2
Figure S2	S3
Figure S3	S4
Figure S4	S5
Table S1	S5
Table S2	S6
Table S3	S6
Table S4	S7
Table S5	S7
Table S6	S8
Table S7	S8
Table S8	S9
Table S9	S10
Table S10	S11
Table S11	S11
Table S12	S12
Table S13	S12
Table S14	S13
Table S15	S13
Table S16	S14
Table S17	S14
Table S18	S15

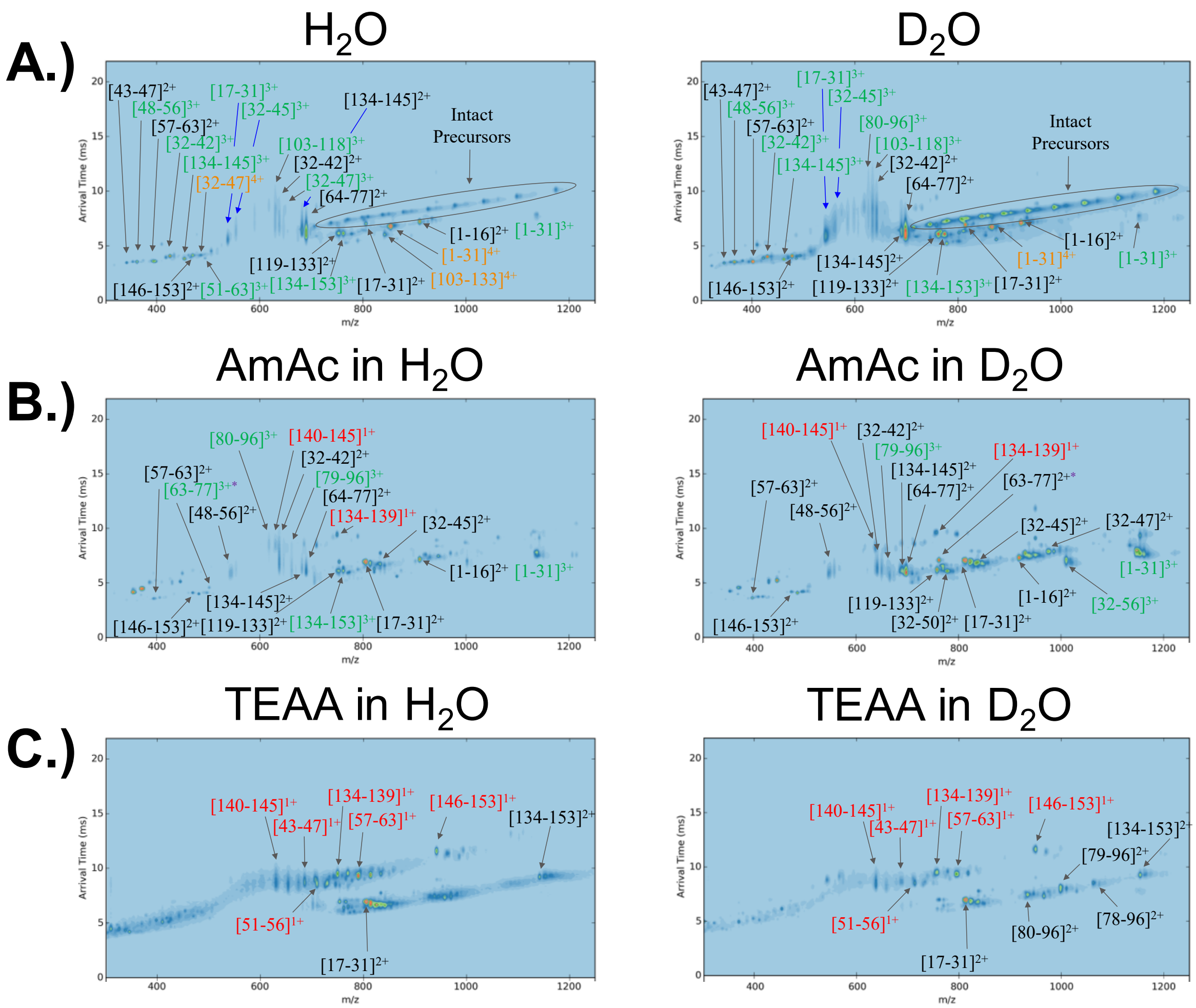

Figure S1. M/z vs arrival time plots of myoglobin digestion products at 21 °C across H_2O and D_2O solutions with all signals labeled, with the addition of A.) no buffers in the solution, B.) AmAc molecules in the solution, and C.) TEAA molecules in the solution. Signals with a purple asterisk (*) could be either peptide 63-77 or peptide 64-78.

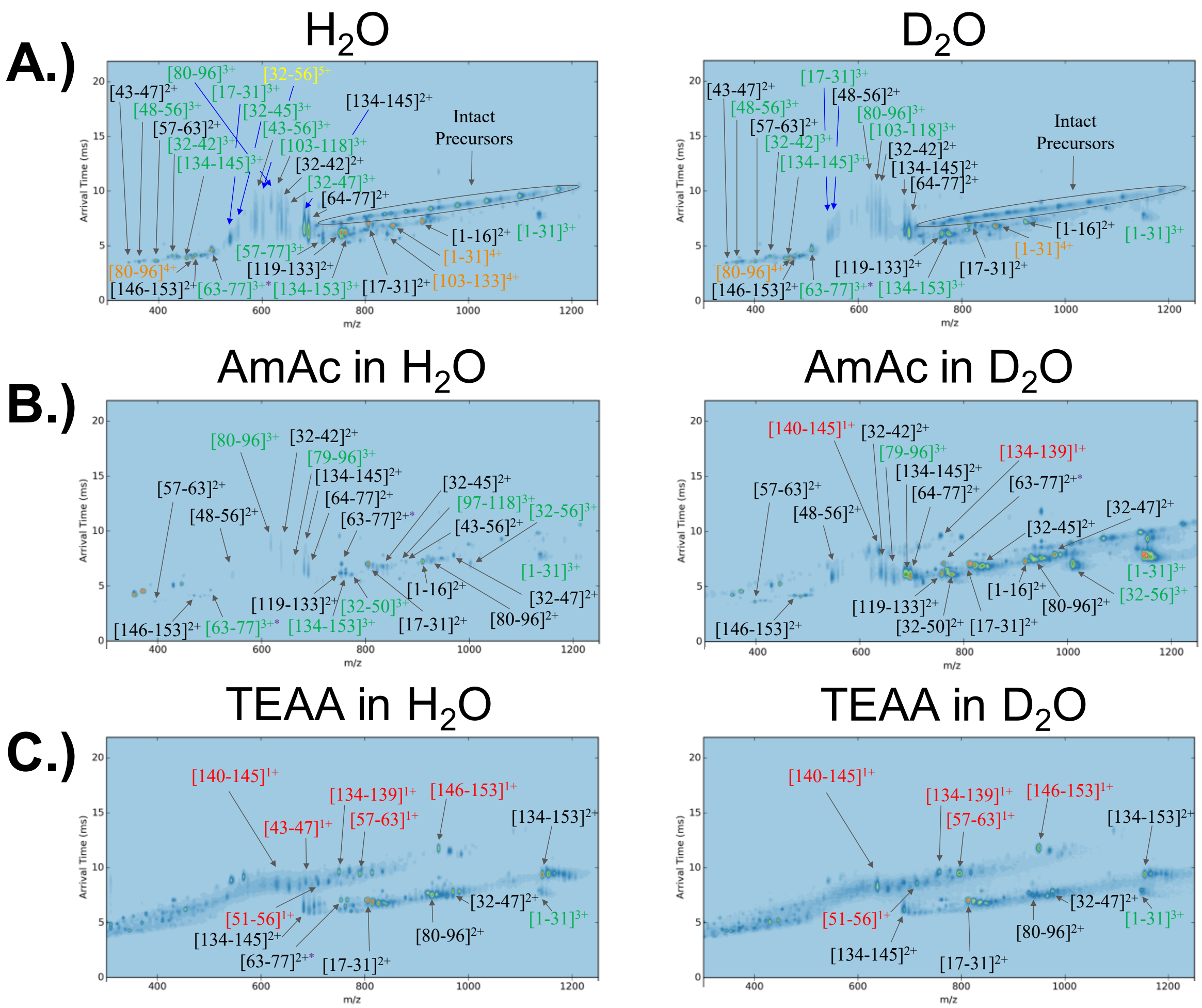

Figure S2. M/z vs arrival time plots of myoglobin digestion products at 4 °C across H_2O and D_2O solutions with all signals labeled, with the addition of A.) no buffers in the solution, B.) AmAc molecules in the solution, and C.) TEAA molecules in the solution. Signals with a purple asterisk (*) could be either peptide 63-77 or peptide 64-78.

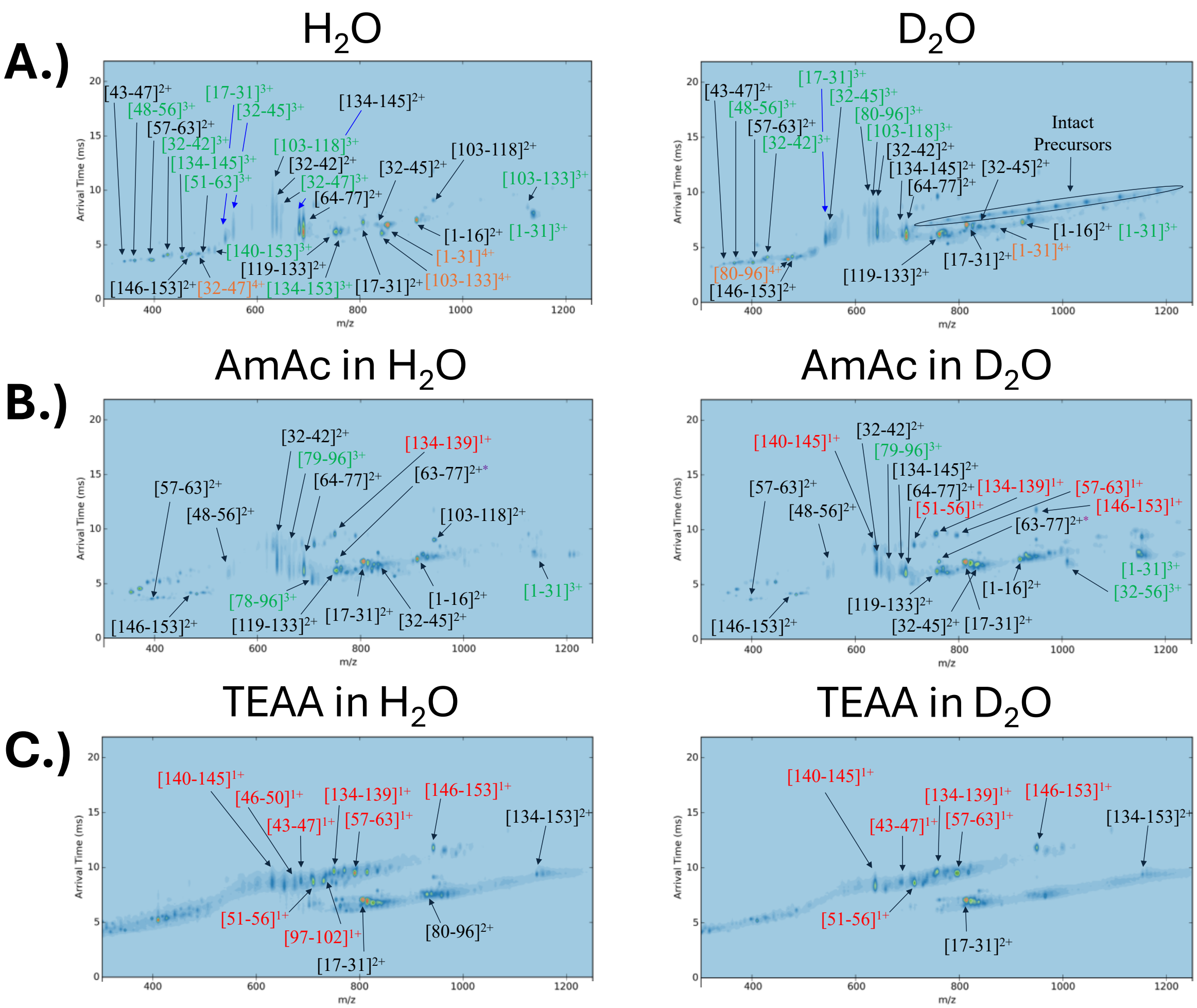

Figure S3. M/z vs arrival time plots of myoglobin digestion products at 37 °C across H_2O and D_2O solutions with all signals labeled, with the addition of A.) no buffers in the solution, B.) AmAc molecules in the solution, and C.) TEAA molecules in the solution. Signals with a purple asterisk (*) could be either peptide 63-77 or peptide 64-78.

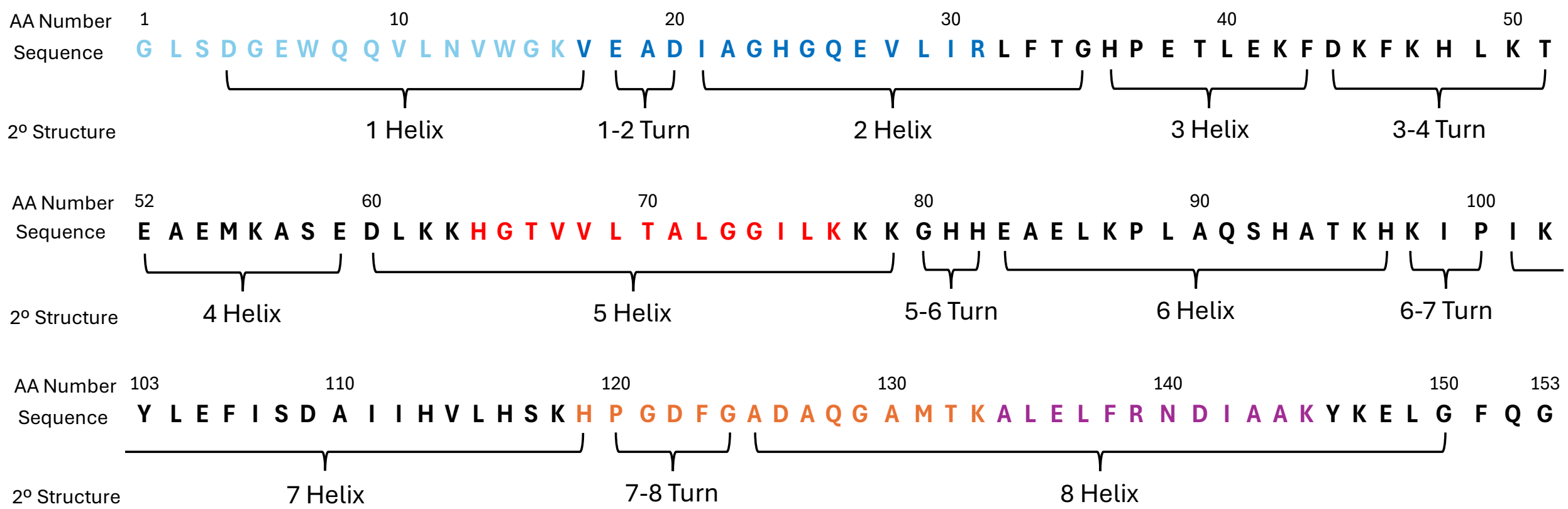

Figure S4: Secondary structure map of myoglobin with peptides identified in main text **Figure 2** highlighted.

Table S1: All Peptides Identified in H2O Solutions Containing No Buffer Molecules at 21 °C

Monoisotopic *m/z*	z	m+H	Sequence	Arrival Time
342.6895	2	684.3717	43-47	3.53
395.7233	2	790.4393	57-63	3.64
471.2474	2	941.4875	146-153	4.19
636.3457	2	1271.6841	32-42	6.84
680.8996	2	1360.7919	134-145	6.39
689.9407	2	1378.8741	64-77	6.17
751.8507	2	1502.6941	119-133	6.17
803.9530	2	1606.8987	17-31	7.06
908.4854	2	1815.9635	1-16	7.28
362.8605	3	1086.5669	48-56	3.53
424.5617	3	1271.6705	32-42	4.08
454.2652	3	1360.7810	134-145	3.86
493.9282	3	1479.7700	51-63	3.86
536.3033	3	1606.8953	17-31	5.51
554.6287	3	1661.8715	32-45	6.28
629.0227	3	1885.0535	103-118	7.28
646.3645	3	1937.0789	32-47	6.50
761.7599	3	2283.2651	134-153	6.17
1135.2820	3	3403.8314	1-31	7.94
485.0210	4	1937.0622	32-47	4.19
842.9498	4	3368.7774	103-133	6.06
851.7172	4	3403.8470	1-31	6.84

**Table S2:** All Peptides Identified in D2O Solutions Containing No Buffer Molecules at 21 °C				
**Base Peak *m/z***	**z**	**m+H**	**Sequence**	**Arrival Time**
345.2026	2	689.3979	43-47	3.42
401.7550	2	802.5027	57-63	3.53
478.2863	2	955.5653	146-153	4.08
642.3808	2	1283.7543	32-42	6.95
691.4572	2	1381.9071	134-145	6.28
697.9786	2	1394.9499	64-77	5.95
760.9035	2	1520.7997	119-133	6.06
814.5105	2	1628.0137	17-31	6.95
922.5587	2	1844.1101	1-16	7.17
367.5554	3	1100.6516	48-56	3.53
428.9233	3	1284.7553	32-42	4.08
461.6366	3	1382.8952	134-145	3.75
544.0043	3	1629.9983	17-31	5.95
560.3260	3	1678.9634	32-45	6.50
627.3464	3	1880.0246	80-96	8.05
635.3867	3	1904.1455	103-118	7.17
773.1555	3	2317.4519	134-153	6.17
1151.3635	3	3452.0759	1-31	7.83
864.2783	4	3454.0914	1-31	6.73

**Table S3:** All Peptides Identified in H2O Solutions Containing 200 mM AmAc at 21 °C				
**Monoisotopic *m/z***	**z**	**m+H**	**Sequence**	**Arrival Time**
631.3272	1	631.3272	140-145	8.38
748.4146	1	748.4146	134-139	9.48
395.7059	2	790.4045	57-63	3.64
471.2284	2	941.4495	146-153	4.08
543.7698	2	1086.5323	48-56	5.95
636.3236	2	1271.6399	32-42	6.84
680.8768	2	1360.7463	134-145	6.39
689.9177	2	1378.8281	64-77	6.06
751.8268	2	1502.6463	119-133	6.17
803.9158	2	1606.8243	17-31	6.95
831.4147	2	1661.8221	32-45	6.84
908.4459	2	1815.8845	1-16	7.17
502.9752	3	1506.9110	63-77 OR 64-78	4.63
618.6505	3	1853.9369	80-96	7.39
661.3427	3	1982.0135	79-96	6.06
761.7358	3	2283.1928	134-153	6.17
1135.2378	3	3403.6988	1-31	7.83

**Table S4:** All Peptides Identified in D2O Solutions Containing 200 mM AmAc at 21 °C				
**Base Peak *m/z***	**z**	**m+H**	**Sequence**	**Arrival Time**
638.3763	1	638.3763	140-145	8.27
399.2355	2	797.4637	57-63	3.64
475.7562	2	950.5051	146-153	4.19
547.8038	2	1094.6003	48-56	5.84
640.3549	2	1279.7025	32-42	6.39
688.4259	2	1375.8445	134-145	6.06
696.4550	2	1391.9027	64-77	5.95
758.8671	2	1516.7269	119-133	6.28
761.5065	2	1522.0057	63-77 OR 64-78	7.17
812.4783	2	1623.9493	17-31	7.06
836.9573	2	1672.9073	32-45	6.95
918.5135	2	1836.0197	1-16	7.39
975.5455	2	1950.0837	32-47	7.94
665.3732	3	1994.1050	79-96	5.62
776.4407	3	2327.3075	32-50	6.17
1009.2277	3	3025.6685	32-56	7.17
1147.6583	3	3440.9603	1-31	8.05

**Table S5:** All Peptides Identified in H2O Solutions Containing 200 mM TEAA at 21 °C				
**Monoisotopic *m/z***	**z**	**m+H**	**Sequence**	**Arrival Time**
631.3382	1	631.3382	140-145	8.60
684.3716	1	684.3716	43-47	8.71
708.3066	1	708.3066	51-56	8.60
748.4266	1	748.4266	134-139	9.59
790.4135	1	790.4135	57-63	9.37
941.4688	1	941.4688	146-153	11.69
803.9158	2	1606.8243	17-31	6.95
1142.0969	2	2283.1865	134-153	9.26

**Table S6:** All Peptides Identified in D2O Solutions Containing 200 mM TEAA at 21 °C				
**Base Peak *m/z***	**z**	**m+H**	**Sequence**	**Arrival Time**
637.3716	1	637.3716	140-145	8.49
687.3940	1	687.3940	43-47	8.60
713.3414	1	713.3414	51-56	8.60
756.4846	1	756.4846	134-139	9.59
795.4481	1	795.4481	57-63	9.37
948.4968	1	948.4968	146-153	11.69
812.4907	2	1623.9741	17-31	7.06
934.5204	2	1868.0335	80-96	7.50
998.5767	2	1996.1461	79-96	8.16
1064.1431	2	2127.2789	78-96	8.60
1153.6489	2	2306.2905	134-153	9.26

**Table S7:** All Peptides Identified in H2O Solutions Containing No Buffer Molecules at 4 °C				
**Monoisotopic *m/z***	**z**	**m+H**	**Sequence**	**Arrival Time**
342.6895	2	684.3717	43-47	3.53
395.7233	2	790.4393	57-63	3.64
471.2474	2	941.4875	146-153	4.19
636.3457	2	1271.6841	32-42	7.28
680.8996	2	1360.7919	134-145	6.50
689.9293	2	1378.8513	64-77	6.17
751.8507	2	1502.6941	119-133	6.17
803.9530	2	1606.8987	17-31	7.06
908.4854	2	1815.9635	1-16	7.28
362.8605	3	1086.5669	48-56	3.64
424.5617	3	1271.6705	32-42	4.08
454.2652	3	1360.7810	134-145	3.86
502.9948	3	1506.9698	63-77 OR 64-78	4.63
536.3033	3	1606.8953	17-31	5.62
554.6390	3	1661.9024	32-45	6.50
584.6531	3	1751.9447	43-56	7.17
618.6505	3	1853.9369	80-96	7.50
629.0227	3	1885.0535	103-118	7.50
646.3645	3	1937.0789	32-47	6.50
717.4453	3	2150.3213	57-77	5.40
761.7599	3	2283.2651	134-153	6.17
1135.2820	3	3403.8314	1-31	7.94
464.2513	4	1853.9834	80-96	3.97
842.9498	4	3368.7774	103-133	6.06
851.7172	4	3403.8470	1-31	6.84
601.7300	5	3004.6209	32-56	6.73

**Table S8:** All Peptides Identified in D2O Solutions Containing No Buffer Molecules at 4 °C				
**Base Peak *m/z***	**z**	**m+H**	**Sequence**	**Arrival Time**
345.7064	2	690.4055	43-47	3.53
401.7550	2	802.5027	57-63	3.64
477.2830	2	953.5587	146-153	4.19
549.3190	2	1097.6307	48-56	6.28
642.3808	2	1283.7543	32-42	7.06
691.4572	2	1381.9071	134-145	6.39
697.9786	2	1394.9499	64-77	6.06
760.9035	2	1520.7997	119-133	6.17
814.5105	2	1628.0137	17-31	7.06
922.5587	2	1844.1101	1-16	7.28
367.5554	3	1100.6516	48-56	3.64
428.9233	3	1284.7553	32-42	4.08
461.3079	3	1381.9091	134-145	3.86
510.0291	3	1528.0727	63-77 OR 64-78	4.63
543.6781	3	1629.0197	17-31	5.95
626.0442	3	1876.1180	80-96	7.61
635.3867	3	1904.1455	103-118	7.28
772.8273	3	2316.4673	134-153	6.17
1151.3784	3	3452.1206	1-31	7.94
469.5314	4	1875.1038	80-96	3.86
864.2783	4	3454.0914	1-31	6.84

**Table S9:** All Peptides Identified in H2O Solutions Containing 200 mM AmAc at 4 °C				
**Monoisotopic *m/z***	**z**	**m+H**	**Sequence**	**Arrival Time**
395.7059	2	790.4045	57-63	3.64
471.2189	2	941.4305	146-153	4.91
543.7800	2	1086.5527	48-56	6.06
636.3236	2	1271.6399	32-42	7.28
680.8768	2	1360.7463	134-145	6.61
689.9177	2	1378.8281	64-77	6.28
751.8268	2	1502.6463	119-133	6.17
753.9618	2	1506.9163	63-77 OR 64-78	7.06
803.9158	2	1606.8243	17-31	7.06
831.4147	2	1661.8221	32-45	6.84
876.4525	2	1751.8977	43-56	7.28
908.4327	2	1815.8581	1-16	7.28
927.4379	2	1853.8685	80-96	7.50
968.9892	2	1936.9711	32-47	7.83
502.9752	3	1506.9110	63-77 OR 64-78	4.63
618.6505	3	1853.9369	80-96	8.38
661.3539	3	1982.0471	79-96	6.39
761.7479	3	2283.2291	134-153	6.17
772.4020	3	2315.1914	32-50	6.06
867.8417	3	2601.5105	97-118	7.39
1002.1715	3	3004.4999	32-56	7.06
1135.2524	3	3403.7426	1-31	7.94

**Table S10:** All Peptides Identified in D2O Solutions Containing 200 mM AmAc at 4 °C				
**Base Peak *m/z***	**z**	**m+H**	**Sequence**	**Arrival Time**
637.3496	1	637.3496	140-145	8.27
398.2143	2	795.4213	57-63	3.64
475.2415	2	949.4757	146-153	4.19
547.2924	2	1093.5775	48-56	5.84
640.3438	2	1279.6803	32-42	6.39
688.4144	2	1375.8215	134-145	6.06
696.4434	2	1391.8795	64-77	5.95
758.3614	2	1515.7155	119-133	6.28
761.4945	2	1521.9817	63-77 OR 64-78	7.17
811.9674	2	1622.9275	17-31	7.17
836.4389	2	1671.8705	32-45	6.95
917.9968	2	1834.9863	1-16	7.39
935.0015	2	1868.9957	80-96	7.61
974.5217	2	1948.0361	32-47	7.94
665.3620	3	1994.0714	79-96	5.73
776.4407	3	2327.3075	32-50	6.17
1008.5474	3	3023.6276	32-56	7.17
1147.3177	3	3439.9385	1-31	8.05

**Table S11:** All Peptides Identified in H2O Solutions Containing 200 mM TEAA at 4 °C				
**Monoisotopic *m/z***	**z**	**m+H**	**Sequence**	**Arrival Time**
631.3272	1	631.3272	140-145	8.49
684.3488	1	684.3488	43-47	8.60
708.3066	1	708.3066	51-56	8.60
748.4146	1	748.4146	134-139	9.70
790.4258	1	790.4258	57-63	9.48
941.4554	1	941.4554	146-153	11.80
680.8654	2	1360.7235	134-145	6.39
753.9618	2	1506.9163	63-77 OR 64-78	7.06
803.9158	2	1606.8243	17-31	7.06
927.4379	2	1853.8685	80-96	7.61
968.9892	2	1936.9711	32-47	7.94
1142.0969	2	2283.1865	134-153	9.37
1135.2524	3	3403.7426	1-31	7.94

**Table S12:** All Peptides Identified in D2O Solutions Containing 200 mM TEAA at 4 °C				
**Base Peak *m/z***	**z**	**m+H**	**Sequence**	**Arrival Time**
637.3716	1	637.3716	140-145	8.38
712.3260	1	712.3260	51-56	8.60
756.4966	1	756.4966	134-139	9.70
796.4717	1	796.4717	57-63	9.48
948.5103	1	948.5103	146-153	11.91
688.4030	2	1375.7987	134-145	6.06
812.9767	2	1624.9461	17-31	7.06
935.0283	2	1869.0493	80-96	7.61
975.0403	2	1949.0733	32-47	7.94
1154.6736	2	2308.3399	134-153	9.48
1147.6879	3	3441.0491	1-31	8.05

**Table S13:** All Peptides Identified in H2O Solutions Containing No Buffer Molecules at 37 °C				
**Monoisotopic *m/z***	**z**	**m+H**	**Sequence**	**Arrival Time**
342.6895	2	684.3717	43-47	3.53
395.7233	2	790.4393	57-63	3.64
471.2474	2	941.4875	146-153	4.19
636.3457	2	1271.6841	32-42	6.95
680.8996	2	1360.7919	134-145	6.39
689.9407	2	1378.8741	64-77	6.17
751.8627	2	1502.7181	119-133	6.17
803.9530	2	1606.8987	17-31	7.06
831.4525	2	1661.8977	32-45	6.95
908.4854	2	1815.9635	1-16	7.28
943.0388	2	1885.0703	103-118	9.15
362.8605	3	1086.5669	48-56	3.64
424.5707	3	1271.6975	32-42	4.08
454.2652	3	1360.7810	134-145	3.86
493.9282	3	1479.7700	51-63	3.86
518.6043	3	1553.7983	140-153	4.63
536.3033	3	1606.8953	17-31	5.62
554.6390	3	1661.9024	32-45	6.28
629.0227	3	1885.0535	103-118	7.17
646.3645	3	1937.0789	32-47	6.73
761.7599	3	2283.2651	134-153	6.17
1123.6025	3	3368.7929	103-133	8.49
1135.2820	3	3403.8314	1-31	7.94
485.0210	4	1937.0622	32-47	4.19
842.9498	4	3368.7774	103-133	6.06
851.7172	4	3403.8470	1-31	6.84

**Table S14:** All Peptides Identified in D2O Solutions Containing No Buffer Molecules at 37 °C				
**Base Peak *m/z***	**z**	**m+H**	**Sequence**	**Arrival Time**
346.2105	2	691.4137	43-47	3.53
401.7550	2	802.5027	57-63	3.64
477.2830	2	953.5587	146-153	4.19
642.3808	2	1283.7543	32-42	6.39
691.4572	2	1381.9071	134-145	6.06
697.9786	2	1394.9499	64-77	5.95
760.9035	2	1520.7997	119-133	6.28
814.5105	2	1628.0137	17-31	7.06
838.9946	2	1676.9819	32-45	6.95
922.5587	2	1844.1101	1-16	7.39
367.5554	3	1100.6516	48-56	3.64
428.5884	3	1283.7506	32-42	4.08
543.3418	3	1628.0108	17-31	5.73
560.3364	3	1678.9946	32-45	6.28
626.0442	3	1876.1180	80-96	6.39
635.3867	3	1904.1455	103-118	6.39
1151.3784	3	3452.1206	1-31	7.94
469.0295	4	1873.0962	80-96	3.97
864.2783	4	3454.0914	1-31	6.95

**Table S15:** All Peptides Identified in H2O Solutions Containing 200 mM AmAc at 37 °C				
**Monoisotopic *m/z***	**z**	**m+H**	**Sequence**	**Arrival Time**
748.4266	1	748.4266	134-139	9.59
395.7059	2	790.4045	57-63	3.64
471.2379	2	941.4685	146-153	4.19
543.7698	2	1086.5323	48-56	5.95
636.3236	2	1271.6399	32-42	7.17
689.9177	2	1378.8281	64-77	6.17
751.8388	2	1502.6703	119-133	6.16
753.9738	2	1506.9403	63-77 OR 64-78	7.06
803.9282	2	1606.8491	17-31	7.06
831.4273	2	1661.8473	32-45	6.84
908.4459	2	1815.8845	1-16	7.28
943.0120	2	1885.0167	103-118	9.04
661.3539	3	1982.0471	79-96	6.17
704.0433	3	2110.1153	78-96	5.18
1135.2378	3	3403.6988	1-31	7.94

**Table S16:** All Peptides Identified in D2O Solutions Containing 200 mM AmAc at 37 °C				
**Base Peak *m/z***	**z**	**m+H**	**Sequence**	**Arrival Time**
638.3763	1	638.3763	140-145	8.38
714.3692	1	714.3692	51-56	8.60
756.4846	1	756.4846	134-139	9.70
796.4594	1	796.4594	57-63	9.48
949.5338	1	949.5338	146-153	11.80
398.2230	2	796.4460	57-63	3.64
475.7657	2	951.5314	146-153	4.19
547.7935	2	1095.5870	48-56	6.17
640.3549	2	1280.7098	32-42	6.50
688.4144	2	1376.8288	134-145	6.06
696.9626	2	1393.9252	64-77	5.95
758.8791	2	1517.7582	119-133	6.28
762.0254	2	1524.0508	63-77 OR 64-78	7.06
812.4783	2	1624.9566	17-31	7.06
836.9573	2	1673.9146	32-45	6.95
918.5135	2	1837.0270	1-16	7.39
665.3845	3	1996.1535	79-96	5.84
1008.5612	3	3025.6836	32-56	7.17
1147.6583	3	3442.9749	1-31	8.05

**Table S17:** All Peptides Identified in H2O Solutions Containing 200 mM TEAA at 37 °C				
**Monoisotopic *m/z***	**z**	**m+H**	**Sequence**	**Arrival Time**
631.3272	1	631.3272	140-145	8.60
672.2921	1	672.2921	46-50	8.38
684.3716	1	684.3716	43-47	8.82
708.3182	1	708.3182	51-56	8.71
735.4717	1	735.4717	97-102	9.37
748.4266	1	748.4266	134-139	9.70
790.4135	1	790.4135	57-63	9.48
941.4554	1	941.4554	146-153	11.80
803.9282	2	1606.8491	17-31	7.06
927.4512	2	1853.8951	80-96	7.61
1142.0969	2	2283.1865	134-153	9.37

**Table S18:** All Peptides Identified in D2O Solutions Containing 200 mM TEAA at 37 °C				
**Base Peak *m/z***	**z**	**m+H**	**Sequence**	**Arrival Time**
637.3827	1	637.3827	140-145	8.38
688.3800	1	688.3800	43-47	8.71
713.3648	1	713.3648	51-56	8.60
756.4846	1	756.4846	134-139	9.70
796.4594	1	796.4594	57-63	9.59
949.5203	1	949.5203	146-153	11.91
812.4783	2	1623.9493	17-31	7.17
1161.6509	2	2322.2945	134-153	9.48
